# Exploring energy geography: Data insights on household consumption

**DOI:** 10.1016/j.dib.2024.110191

**Published:** 2024-02-21

**Authors:** Bardia Mashhoodi, Thijs Bouman

**Affiliations:** a Landscape Architecture and Spatial Planning Group, Department of Environmental Sciences, Wageningen University & Research, P.O. box 47, 6700 AA Wageningen, the Netherlands; b Faculty of Behavioural and Social Sciences, University of Groningen, the Netherlands

**Keywords:** GIS, Energy demand, Energy geography, Energy poverty, Geographically weighted regression, Spatial analysis

## Abstract

Household energy consumption (HEC) varies across neighbourhoods and gender groups. This database provides raw and analysed data on HEC determinants and their estimated influence on HEC in 2707 residential neighbourhoods (Wijk) in the Netherlands in 2018. The raw data consists of 17 indicators on energy demand, socioeconomic characteristics, microclimate and buildings. The indicators are retrieved from and calculated based on open national and international datasets. The analysed data presents the local coefficients of the HEC determinants, the outcome of the geographically weighted regression model (GWR) presented in the related article [1].

Specifications Table

**Table:** 

Subject	Energy
Specific subject area	Household energy consumption
Type of data	GIS data (.shp; .tif); text (.txt); SPSS (.sav); Excel (.xlsx) Raw and analysed.
Data collection	The raw data are retrieved from three Dutch sources (CBS, 3d BAG and KNMI), an EU source (Copernicus), and NASA satellite imagery.
Data source location	The Netherlands (52 °22 N 4 °53 E) The primary sources are retrieved from: Statistics Netherlands (Centraal Bureau voor de Statistiek): Wijk- en buurtkaart 2018, 2018. https://www.cbs.nl/nl-nl/dossier/nederland-regionaal/geografische-data/wijk-en-buurtkaart-2018. (Accessed 26 June 2018). Esri Netherlands: 3D BAG, http://www.esri.nl/nl-NL/news/nieuws/sectoren/nieuw-in-arcgis-voor-leefomgeving Royal Netherlands Meteorological Institute (KNMI): http://www.sciamachy-validation.org/climatology/daily_data/selection.cgi European Environment Agency: https://www.eea.europa.eu/data-and-maps/data/clc-2012-raster NASA Earthdata: https://earthdata.nasa.gov/.
Data accessibility	Repository name: Mendeley Data identification number (DOI): 10.17632/9t76sxgm5f.4 Direct URL to data: https://data.mendeley.com/datasets/9t76sxgm5f/4 Instructions for accessing these data: For the complete dataset, use “version 4” via the link above.
Related research article	Mashhoodi, B. and Bouman, T., 2023. Gendered geography of energy consumption in the Netherlands. Applied Geography, 154, p.102936. 10.1016/j.apgeog.2023.102936

## 1 Value of the Data

• The dataset offers a comprehensive set of high-resolution, geo-referenced data and estimated impacts on HEC to study household energy consumption, environmental inequality, and energy poverty.
• Researchers in human geography, spatial planning, energy, and environmental studies can use the database.
• The data can be further developed for studying climate change, environmental hazards and electric mobility.

## 2 Background

The database is produced to identify and map the gender groups with the highest energy demand in Dutch neighborhoods. The database includes geo-referenced socioeconomic, housing, environmental, and land cover data. This article paves the way for a comprehensive study at the scale of Dutch neighbourhoods (similar to the original article [1]) and finer scales.

## 3 Data Description

### 3.1 Raw datasets

The presented database is retrieved, calculated, and aggregated using six open-to-public data sources stored in the “Raw” folder.

#### 3.1.1 Neighborhood census

Published by the Dutch central bureau for Statistics, Wijk-en-buurtkaart 2018 provides the annual census on gas and electricity consumption and socioeconomic characteristics of neighborhoods [2]. The data is saved in the folder “Raw\WijkBuurtkaart_2018_v3”.

#### 3.1.2 Building database

The 3D BAG dataset provides GIS data on the buildingsʼ shape, location, height, and construction year [3]. The raw data is stored in raster format: height of buildings (Raw\Buildings\Buildings_Age) and age of buildings (Raw\Buildings\Buildings_DEM).

#### 3.1.3 Meteorological data

The records of air temperature (Raw\KNMI_2018\CDD_HDD\KNMIStations_TG_TN_TX_2018), humidity at 1.5 m height (Raw\KNMI_2018\Humidity\KNMI_humidity_2018) and wind at 10 m height (Raw\KNMI_2018\Wind\KNMI Wind FG 2018) at the KNMI meteorological stations in 2018 [4].

#### 3.1.4 Land cover data

To estimate aerodynamic roughness length and wind speed using KNMI data, the CORINE land cover 2018 is used (Raw\CORINE_raster100m\CORINE_raster100m) [5].

#### 3.1.5 Land surface temperature

The nine MODIS/Terra and nine MODIS/Aqua, each representing an eight-day average land surface temperature for specific periods, are retrieved and stored (Raw\LST_MOD11A2_MYD11A2) [6].

#### 3.1.6 Normalized difference vegetation index (NDVI)

The annual NDVI data represents the average monthly MODIS/Terra Vegetation Indices Monthly L3 Global 1km SIN Grid V006 (Raw\NDVI) [6].

### 3.2 Interopolated meteorological rasters

The observations of the KNMI stations and satellite imagery are interpolated and aggregated. (For a detailed description of data processing, see the original publication [1].) The results are stored in five geo-referenced raster files in the “Interpolated_rasters\ Final_Rasters.gdb” folder: CDD_2018; HDD_2018; Humidity2018; NDVI2018; Wind_2018.

### 3.3 Dataset aggregated at the scale of Dutch residential zones

The final database aggregates all the above data at the scale of neighbourhoods in the Netherlands (Aggregated\ Final_database.sav). Table 1 shows the acronyms and descriptions of the database variables.

**Table 1:** The description of data in the neighborhood-aggregated database.

Variable acronym	Description
wk_code	Neighborhood code, designated by CBS [2].
X	Longitude of neighborhood's centroid with RD NEW coordinate system
Y	Latitude of neighborhood's centroid with RD NEW coordinate system
HEC	The total gas and electricity consumption per head (MJ) in 2018 (Find the map in Ref. [1])
Female	Percentage of female population minus the percentage of male population
gem_hh_gr	Average household size
p_laaginkp	Percentage of low-income households
p_hooginkp	Percentage of high-income households
p_00_14_jr	Percentage of the population aged 14 years or younger
p_15_24_jr	Percentage of the population aged 15–24 years
p_65_eo_jr	Percentage of the population aged 65 or older
P_Immigrants	Percentage of the population with at least one foreign parent.
p_huurwon	Percentage of rental dwellings
Bui_Meadian_Age	Median building age, weighted by gross floor area
S_to_V	The ratio of buildings’ surfaces to buildings’ volumes
Humidity	Humidity percentage at 1.5 height
Wind	Wind speed at 10 m height (km/hr)
HDD	Heating Degree days (for the definition, see [1])
CDD	Cooling Degree days (for the definition, see [1])
NDVI	Normalized difference vegetation index summer 2018
LSTDay	Average land surface temperature at 10:30 a.m. and 1:30 p.m. in summer 2018 (°C)
LSTNight	Average land surface temperature at 10:30 p.m. and 1:30 a.m. in summer 2018 (°C)

### 3.4 Estimates of the GWR Model

The final database (GWR Estimates\GWR_Adaptive_Bisquare_band380.xlsx) contains estimates of the GWR model for each residential zone (for specification of the model, see [1]). The dependent variable of the GWR model is annual HEC per head. The estimates show the impact (i.e. standardised coefficient) of the socioeconomic, building characteristics, meteorological factors, and land cover on the HEC of each residential zone. The fields in the files are as follows:

• Wijk_code: Residential zone's identification code, corresponding with the raw data from CBS [2].
• x_coord: x coordinate of the residential zone's centroid with RD New projection
• y_coord: x coordinate of the residential zone's centroid with RD New projection
• est_intercept: the estimate of the constant term at the Wijk
• se_intercept: standard error of the constant term at the Wijk
• t_intercept: pseudo t value (estimate / standard error) the constant term at the Wijk
• est_Z*: the estimated standardised coefficient of variable * (see Table 1) at the Wijk
• se_ Z*: standard error of the estimated standardised coefficient of variable * (see Table 1) at the Wijk
• t_ Z*: pseudo t value (estimate / standard error) the estimated standardised coefficient of the variable * (see Table 1) at the Wijk
• residual: standardised residual of the GWR model at the wijk
• localR2: R2 value of the GWR model at the wijk

The GWR model also estimates the local standardised coefficients of the interaction terms between gender and other socioeconomic variables. The interaction terms are marked by “X” in the middle of the variables’ names. For instance, est_ZFemaleXBui_Meadian_Age shows the estimated standardised coefficient of the interaction between gender (Female) and building age.

## 4 Experimental Design, Materials and Methods

In the first step, the data on the residential zones on energy consumption and socioeconomic characteristics are retrieved. The data is in the format of polygons (.shp). The residential zones with a missing piece of data, presumably non-residential, are excluded from the database. In the second step, other data types are retrieved or interpolated as raster data. Subsequently, the raster data are aggregated at the residential zones, and the final aggregated database is obtained. At the last stage, using the Golden Selection Search function of the GWR4 software [7], the best bandwidth for the application of a GWR model is found, and the local coefficients are estimated (for more details, see [1]).

The data presented in this article provides a comprehensive set of determinants for analysing household energy consumption at the neighbourhood scale. The provision of data on gas and electricity demand allows for geographic analysis of different energy sources (similar to [7]) and the influence of urban morphology on energy demand (similar to [8]). Furthermore, it paves the way for the analysis of environmental impacts, such as land surface temperature, on energy demand (similar to [9]) and environmental inequality among socioeconomic groups (similar to [10,11]). The database provides the basis for geographic studies on energy poverty (similar to [12,17]) and households’ energy expenditure (similar to [13]). Using the database, further studies can estimate the energy grid congestion (similar to [14]) and optimally allocate new points for energy supply to meet the upcoming charging demand of electric vehicles (similar to [15,16]).

## Limitations

Not applicable.

## Ethics Statement

The raw data of this study is provided by open, public GIS data sources, in full compliance with ethical requirements for publication in the journal of Data in Brief.

## CRediT authorship contribution statement

**Bardia Mashhoodi:** Conceptualization, Methodology, Software, Data curation, Writing – original draft, Visualization, Investigation, Validation, Writing – review & editing. **Thijs Bouman:** Conceptualization.

## Data Availability

Studying household energy consumption: 19 essential spatial data and their estimated impacts (Original data) (Mendeley Data).
